# Supplementary material for: Covid-19 vaccine uptake and its associated factors among adult population in Dangila district, Awi Zone, Northwest Ethiopia: A mixed method study
Source: PLoS One. 2024 May 14;19(5):e0302531. doi: 10.1371/journal.pone.0302531 (PMC11093357; doi:10.1371/journal.pone.0302531)
Supplement: S1 Table — (DOCX) [file pone.0302531.s002.docx]

Supplementary Table 1: COVID-19 related knowledge of study subjects

| S/N | **Questions** | **Yes (%)** | **No (%)** |
| --- | --- | --- | --- |
| 1 | Do hear about COVID vaccine. | 540 (90%) | 60 (10%) |
| 2 | COVID-19 can be prevented by vaccination. | 490 (81.7%) | 110 (18.3%) |
| 3 | COVID 19 vaccines produce long term-immunity | 386 (64.3%) | 214 (35.7%) |
| 4 | COVID 19 vaccines can reduce disease severity | 484 (80.7%) | 114 (19.3%) |
| 5 | COVID 19 vaccine may have adverse effects | 459 (76.5%) | 141 (23.5%) |
| 6 | Do you know the types of COVID 19 vaccines currently given in Ethiopia? | 136 (22.7%) | 464 (77.3) |
| 7 | If Q307 is “yes” Which types of COVID 19 vaccines currently given in Ethiopia? |  | Frequency (%) |
|  |  | 1. Pfizer | 6 (1%) |
|  |  | B. Janssen | 28 (4.7%) |
|  |  | c. Astrazenaca | 72 (12%) |
|  |  | D. Other (specify)—“I forget the name of vaccines” | 30 (22%) |
| 8 | Do you know the safety of COVID 19 vaccine | 235(39.17%) | 365(60.83%) |
| 9 | COVID-19 vaccine causes allergic reactions | 399(66.5%) | 201 (33.5%) |
| 10 | Are COVID-19 vaccines accessible for all population | 400(66.7%) | 200 ((33.3) |
