# Supplementary material for: Covid-19 vaccine uptake and its associated factors among adult population in Dangila district, Awi Zone, Northwest Ethiopia: A mixed method study
Source: PLoS One. 2024 May 14;19(5):e0302531. doi: 10.1371/journal.pone.0302531 (PMC11093357; doi:10.1371/journal.pone.0302531)
Supplement: S2 Table — (DOCX) [file pone.0302531.s003.docx]

Supplementary Table 2: Attitude of study subjects towards COVID-19 vaccine

| S/N | **Questions** | Strongly Agree | Agree | Neutral | Disagree | Strongly disagree |
| --- | --- | --- | --- | --- | --- | --- |
| 1 | COVID-19 vaccination is essential to control the pandemic | 42(7.0%) | 379(63.2%) | 47(7.8%) | 88(14.7%) | 44(7.3%) |
| 2 | COVID 19 vaccine reduces the chance of getting COVID-19. | 73(12.1%) | 354(59.0%) | 67(11.2%) | 96(16.0%) | 10(1.7%) |
| 3 | Vaccination lowers the risks of COVID-19 complication? | 44(7.3%) | 291(48.5%) | 113(18.8%) | 137(22.8%) | 15(2.5%) |
| 4 | The information given by official media on COVID 19 vaccine is trustable. | 56(9.3%) | 364(60.7% | 70(11.7%) | 96(16%) | 14(2.3%) |
| 5 | Health professionals’ advices are reliable. | 71(11.9) | 422(70.3%) | 39(6.5%) | 54(9.0%) | 14(2.3%) |
| 6 | The current COVID-19 vaccine is safe. | 55(9.1%) | 289(48.2%) | 157(26.2%) | 87(14.5%) | 12(2.0%) |
| 7 | The current COVID-19 vaccine is effective | 46(7.7%) | 305(50.8%) | 130(21.7%) | 107(17.8% | 12(2.0%) |
| 8 | COVID-19 vaccination should be mandatory. | 57(9.5%) | 172(28.7%) | 41(6.8%) | 305(50.8%) | 25(4.2%) |
| 9 | COVID-19 vaccine can reduce the spread of the virus in the community | 52(8.7%) | 322(53.7%) | 103(17.2%) | 111(18.5%) | 12(2.0%) |
| 10 | It is not possible to reduce the incidence of COVID-19 without vaccination | 31(5.2%) | 230(38.3%) | 108(18.0%) | 202(33.7%) | 29(4.8%) |
