# Supplementary material for: Covid-19 vaccine uptake and its associated factors among adult population in Dangila district, Awi Zone, Northwest Ethiopia: A mixed method study
Source: PLoS One. 2024 May 14;19(5):e0302531. doi: 10.1371/journal.pone.0302531 (PMC11093357; doi:10.1371/journal.pone.0302531)
Supplement: Attachment — Submitted filename: A rebuttal letter.docx [file pone.0302531.s004.docx]

**A rebuttal letter**

Journal name: PLOS ONE

PONE-D-23-08920

**Title: Covid-19 vaccine uptake and its associated factors among adult population in Dangila district, Awi Zone, Northwest Ethiopia: A semi-qualitative cross sectional study**

| **Editor’s comments** | **Authors’ response** |
| --- | --- |
| When submitting your revision, we need you to address these additional requirements.  1. Please ensure that your manuscript meets PLOS ONE's style requirements, including those for file naming. The PLOS ONE style templates can be found at  https://journals.plos.org/plosone/s/file?id=wjVg/PLOSOne_formatting_sample_main_body.pdf and  https://journals.plos.org/plosone/s/file?id=ba62/PLOSOne_formatting_sample_title_authors_affiliations.pdf | Thank you dear editor for your valuable comment. The revised manuscript is revised for the PLOS ONE’s style requirements. |
| 2. We noticed you have some minor occurrence of overlapping text with the following previous publication(s), which needs to be addressed:  - https://doi.org/10.1177/20503121221077585  - https://doi.org/10.2147/IDR.S360103  - https://doi.org/10.3389/fpubh.2022.919494  In your revision ensure you cite all your sources (including your own works), and quote or rephrase any duplicated text outside the methods section. Further consideration is dependent on these concerns being addressed. | Thank you dear editor for your comment. As per your comment, the revised manuscript has been edited for the overlapping texts. |
| 3. We note that Figure 1 in your submission contain map/satellite images which may be copyrighted. All PLOS content is published under the Creative Commons Attribution License (CC BY 4.0), which means that the manuscript, images, and Supporting Information files will be freely available online, and any third party is permitted to access, download, copy, distribute, and use these materials in any way, even commercially, with proper attribution. For these reasons, we cannot publish previously copyrighted maps or satellite images created using proprietary data, such as Google software (Google Maps, Street View, and Earth). For more information, see our copyright guidelines: http://journals.plos.org/plosone/s/licenses-and-copyright.    We require you to either (1) present written permission from the copyright holder to publish these figures specifically under the CC BY 4.0 license, or (2) remove the figures from your submission:    a. You may seek permission from the original copyright holder of Figure 1 to publish the content specifically under the CC BY 4.0 license.    We recommend that you contact the original copyright holder with the Content Permission Form (http://journals.plos.org/plosone/s/file?id=7c09/content-permission-form.pdf) and the following text:  “I request permission for the open-access journal PLOS ONE to publish XXX under the Creative Commons Attribution License (CCAL) CC BY 4.0 (http://creativecommons.org/licenses/by/4.0/). Please be aware that this license allows unrestricted use and distribution, even commercially, by third parties. Please reply and provide explicit written permission to publish XXX under a CC BY license and complete the attached form.”    Please upload the completed Content Permission Form or other proof of granted permissions as an ""Other"" file with your submission.    In the figure caption of the copyrighted figure, please include the following text: “Reprinted from [ref] under a CC BY license, with permission from [name of publisher], original copyright [original copyright year].”    b. If you are unable to obtain permission from the original copyright holder to publish these figures under the CC BY 4.0 license or if the copyright holder’s requirements are incompatible with the CC BY 4.0 license, please either i) remove the figure or ii) supply a replacement figure that complies with the CC BY 4.0 license. Please check copyright information on all replacement figures and update the figure caption with source information. If applicable, please specify in the figure caption text when a figure is similar but not identical to the original image and is therefore for illustrative purposes only.  The following resources for replacing copyrighted map figures may be helpful:    USGS National Map Viewer (public domain): http://viewer.nationalmap.gov/viewer/  The Gateway to Astronaut Photography of Earth (public domain): http://eol.jsc.nasa.gov/sseop/clickmap/  Maps at the CIA (public domain): https://www.cia.gov/library/publications/the-world-factbook/index.html and https://www.cia.gov/library/publications/cia-maps-publications/index.html  NASA Earth Observatory (public domain): http://earthobservatory.nasa.gov/  Landsat: http://landsat.visibleearth.nasa.gov/  USGS EROS (Earth Resources Observatory and Science (EROS) Center) (public domain): http://eros.usgs.gov/#  Natural Earth (public domain): http://www.naturalearthdata.com/ | Thank you so much dear editor for your valuable comment. As per your comment, the figure has been removed from the revised manuscript for it has no a significant input for the study. |
| 4. We are unable to open your Supporting Information file S1 File.sav. Please kindly revise as necessary and re-upload. | Thank you for the comments. The S1 File has been re-uploaded with the revised manuscript after checking for its functionality. |
| 5. Please include captions for your Supporting Information files at the end of your manuscript, and update any in-text citations to match accordingly. Please see our Supporting Information guidelines for more information: http://journals.plos.org/plosone/s/supporting-information.  **Comments from Reviewer #1** | Thank you for your comment. As per your comment, the necessary amendment has been made in the revised manuscript. |
| Thank you this intersting piece of work on covid vaccine uptake from Ethiopia  The following key comments:  1. Integration of methods, data, findings ans conclusions is a key challenge of this work. The authors faced a great dealnof challenge to tringulate the qualitative and quantitative methodd. The findings of both methods should be mixed as appropriate instead of being put as separate pieces. So, please integrate the finding. This would pave ways to draw appropriate conclusion and and improve assimilation of findings. | Thank you dear reviewer for your interesting comment. The findings of the two methods were presented sequentially, for the study was sequential mixed method. As per your comment we have refined the triangulation of the findings in the revised manuscript. |
| **2.** Qualitative methods seem overlooked. First ly, though saturation is mentioned, there is no adequate internal evidence that saturation is reached. 1. Tentative sample sizes were not mentioned, 2. Data triangulation is not well indicated. Secondly, data analysis and rigor is not well stated. What type of thematic analysis and coding was applied- open or priori codinf? The procedure paased through transcription, coding, and analysis overly depended on one person, the principal investigator. That is not auditable, not based on peer debriefying etc. So please mention the roles of othet authors in the qualiative process. Please indicate the extent to which subjective neitrality, refxivity, tick description, etc was ensured toward dependebality of the interpretations. Please revise and improve this aspect of the study. | Thank you dear reviewer for your constructive comments. As per your comments, the revised manuscript has been revised and edited for the qualitative component, and the changes have been indicated with track changes. The issues raised in this comment were addressed in page 9 of the revised manuscript and changes have been indicated by highlights. “The investigators ensured that the study was trustworthy based on Lincoln and Cuba’s criteria of credibility, dependability, conformability, and transferability. To maintain the credibility of the research findings, the study participants were observed persistently at the time of the interview. Peer-debriefing was done for the questioner. Dependability was attained through accurate documentation by minimizing spelling errors through frequently observing data and including all documents in the final report, such as including the notes written during the interview and ensuring that the details of the procedure was described to allow the readers to see the basis upon which conclusions were made. To achieve conformability of the study findings, raw data, interview and observation notes, documents and records collected from the field, and others were documented for cross checking. To maintain the transferability of the finding, appropriate probes was used to obtain detailed information on responses, and study participants were selected based on their specific purpose to answer study questions and to get greater in-depth findings” |
| **3.** In the abstract you mentioned..." trascribed reduced, coded, ......". This should be revised. Coding should precede reductioj. Thematazation is the process of data reduction, but coding should be first. | Thank you dear reviewer for your constructive comments. As per your comment, the necessary amendment has been made in the revised manuscript. |
| 4. Measurements lack attitide, beleifs.....which is limitation | Thank you dear reviewer for your constructive comments. The attitude and knowledge of respondents were assessed in the quantitative component of the study and the findings were presented both in text and in table 3, and located in page 12 of the revised manuscript. |
| 5. Was qualitative and quantitative parellel or sequnatial? Why? Did you use qualitative findings to develop quantitative tools? Ypu shoild have done that. So, the authors should improve on integration of findings and qualiative methods for proper conclusion. | Thank you dear reviewer for your constructive comments. The study was sequential mixed method. In this case we analyzed the qualitative part after the quantitative analysis was made. Hence, the revised manuscript has been edited in that way. |
| **Comments from Reviewer #2** |  |
| Dear Authors, thank you for your hard work and public health important manuscript. Although, it is a  good research output, a lot remains to be done to bring it to the expected quality both technically and  grammatically with due care before it gets to the stage of publishing as aimed.  Thus, you are strongly advised to go through all the given comments and inquiries in here with attached  PDF one by one; revise your whole manuscript to bring it to the level of expected quality both  technically and grammatically towards the positive decision | Thank you dear reviewer for your constructive comments. The revised manuscript has been edited for technical and grammatical errors. Besides, all comments of the reviewers and editors have been corrected and indicated by track changes in the revised manuscript. |
