# Supplementary material for: Covid-19 vaccine uptake and its associated factors among adult population in Dangila district, Awi Zone, Northwest Ethiopia: A mixed method study
Source: PLoS One. 2024 May 14;19(5):e0302531. doi: 10.1371/journal.pone.0302531 (PMC11093357; doi:10.1371/journal.pone.0302531)
Supplement: Attachment — Submitted filename: A rebuttal letter 2.docx [file pone.0302531.s005.docx]

**A rebuttal letter 2**

Journal name: PLOS ONE

PONE-D-23-08920

**Title: Covid-19 vaccine uptake and its associated factors among adult population in Dangila district, Awi Zone, Northwest Ethiopia: A semi-qualitative cross sectional study**

| **Editor’s comments** | **Authors’ response** |
| --- | --- |
| 1. Please review your reference list to ensure that it is complete and correct. If you have cited papers that have been retracted, please include the rationale for doing so in the manuscript text, or remove these references and replace them with relevant current references. Any changes to the reference list should be mentioned in the rebuttal letter that accompanies your revised manuscript. If you need to cite a retracted article, indicate the article’s retracted status in the References list and also include a citation and full reference for the retraction notice. | Thank you dear editor for your valuable comment. The revised manuscript is revised for the presence of retracted references. We found that none of them are retracted. |
| **Comments from Reviewer #2** |  |
| Dear Author, thank you for sending your improved version of the manuscript on COVID-19 Vaccine up take and associated factors . Although you tried to address many comments forwarded from the reviewers ,still the problem of addressing clearly the objective and expected contents of a mixed methods approach is not clearly addressed   1. In addition, in your title section you said it was a <>study design and in methodology section you said it is a mixed method design. Thus, it is good to be consistent. | Thank you dear reviewer for your constructive comments. As per your comment, the revised manuscript is edited for consistency in terms of terminologies in the design both in the title and methodology. |
| 2. Most importantly in your discussion section the concept of triangulation or support of quantitative data with that of qualitative is still not written to the expected standard or quality . | Thank you dear reviewer for your constructive comments. As per your comment we have included the following paragraph in the discussion section of the revised manuscript. The detail of the qualitative finding analysed sequentially has been written in the result part. Methdological aspects have been also written in the methodology part.  “The quantitative findings were further validated through a meticulous analysis of the qualitative data. Thematic exploration concerning barriers to COVID-19 vaccine acceptance among the adult population in Dangila district, Awi Zone, revealed three primary themes, each with seven sub-themes. These included Vaccine-related factors, Personal factors, and Environmental factors, encompassing issues such as the Availability of vaccine, Misperceptions about vaccine efficacy and safety, Lack of knowledge about the COVID-19 vaccine, Mis trust, Fear of adverse effects, Social media influence, and Religious beliefs. Notably, these qualitative insights mirror the patterns observed in the quantitative analysis, affirming the robustness and consistency of the findings across both methodologies.” |
| 3. Be it sequential or not , I suppose, it should be clearly articulated or shown in such a way that whether a finding of a method was supported or not by the other data, source or method during triangulation for better validity of the finding/s rather than giving each of them in independent sections. | Yes dear reviewer, we totally agreed with this and hence, we have stated this triangulation and consistency of findings of the two methods in the discussion part and it has been indicated in the revised manuscript with track changes. Thank you for your efforts to improve our manuscript. |
