# Supplementary material for: Covid-19 vaccine uptake and its associated factors among adult population in Dangila district, Awi Zone, Northwest Ethiopia: A mixed method study
Source: PLoS One. 2024 May 14;19(5):e0302531. doi: 10.1371/journal.pone.0302531 (PMC11093357; doi:10.1371/journal.pone.0302531)
Supplement: Attachment — Submitted filename: A rebuttal letter 3.docx [file pone.0302531.s006.docx]

**A rebuttal letter 3**

Journal name: PLOS ONE

PONE-D-23-08920

**Title: Covid-19 vaccine uptake and its associated factors among adult population in Dangila district, Awi Zone, Northwest Ethiopia: A mixed method study**

| **Editor’s comments** | **Authors’ response** | |
| --- | --- | --- |
| Journal Requirements:  Please review your reference list to ensure that it is complete and correct. If you have cited papers that have been retracted, please include the rationale for doing so in the manuscript text, or remove these references and replace them with relevant current references. Any changes to the reference list should be mentioned in the rebuttal letter that accompanies your revised manuscript. If you need to cite a retracted article, indicate the article’s retracted status in the References list and also include a citation and full reference for the retraction notice. | Thank you dear editor for your valuable comment. The revised manuscript is revised for the presence of retracted references. We found that none of them are retracted. | |
| **Comments from reviewer #1** | | |
| Thank you for inviting me to review this interesting work. I will provide my general remark on the manuscript as follows:  1. I know that it is not necessary to publish on PLOS ONE when a study has only few finding. This study has not added to existing literature, even in Ethiopia.  2. It is great that this is a mixed-method study. I would suggest the authors to specifically mention which type of mixed-method is this? Is it convergent/parallel or explanatory sequential or exploratory sequential one? The problem, such a study should have benefited a lot from exploratory sequential mixed-method. There may be many local and cultural beliefs and influences specific to Dangila that the authors could have explored qualitatively first then provide quantitative evidence latter (this can be a convincing research question to be asked at this time). | | Absolutely, I completely understand your perspective. While this study may not seem groundbreaking at first glance, it actually addresses a crucial issue: the low COVID-19 vaccine uptake in the region. By employing a mixed-method, community-based approach, this study likely offers valuable insights and potential strategies for improving vaccine acceptance and uptake. Despite its seemingly limited scope, its potential impact on public health in Ethiopia cannot be overlooked.  The mixed method study employed was an explanatory sequential mixed-method. We preferred this approach to clarify or elaborate on quantitative findings by exploring participants' perspectives or contexts. |
| 3. Again another problem exists analysis approach of this mixed-method. As it currently stands, the authors did not triangulate the quantitative and qualitative findings. Each finding appeared stand-alone- this means the findings are not assimilated well and not ready for discussion. Please revise. Don't have knowledge, attitude....titles twice in this same manuscript. If you start describing or explaining about knowledge about COVID-19 or its vaccine, please triangulate both qualitative and quantitative findings in that same sub-section. You should not come again explaining about knowledge qualitatively or quantitatively. Same holds true with other reported sub-titles such as attitude or social influences. | | Thank you dear reviewer for your constructive comments. As per your suggestion, the revised manuscript is writing the quantitative and qualitative findings together with proper triangulation. The changes made have been shown with track changes in the revised manuscript. |
| 4. Please say "religious beliefs" instead of "religious believe". please check such issues throughout the manuscript. You may use freely available softwares such as Grammarly or other support centers. | | Thank you dear reviewer for your comment. The necessary correction has been made in the revised manuscript. |
| 5. Measurement and analysis of knowledge is not exhaustive and convincing. | |  |
| 6. Using mean to categorize knowledge and or attitude into poor and good is unnecessary nomination or judgement of the respondents- use standard reference to category these. Our use quartiles or standardized mean to report in the form of proportion or use count of basic knowable knowledge items by referring to WHO or national expectations. You can reply on expert discussion or opinion to identify which are the minimum knowable items, otherwise. | | Thank you for your constructive comments. The measurement of knowledge and attitude has been indicated in the methodology section of the revised manuscript with citation of the appropriate reference. |
| 7. Before moving on to discussion, please make proper triangulation of both qualitative and quantitative data | | Thank you dear reviewer for your constructive comments. As per your comment, the necessary correction has been made in the revised manuscript. |
| 8. Qualitative findings are shallow. Please immerse yourself with the data and provide tick description. | | Thank you for your comments. Some modifications have been made in the revised manuscript. |
| 9. Provide has detail techniques through which you assured credibility, dependability, transferability and confirmability of qualitative findings. | | Thank you for your constructive comment. We included the following paragraph in the methodology section. “We ensured that the study was trustworthy based on Lincoln and Cuba’s criteria of credibility, dependability, conformability, and transferability. To maintain the credibility of the research findings, the study participants were observed persistently at the time of the interview. Peer-debriefing was done for the questioner. Dependability was attained through accurate documentation by minimizing spelling errors through frequently observing data and including all documents in the final report, such as including the notes written during the interview and ensuring that the details of the procedure was described to allow the readers to see the basis upon which conclusions were made. To achieve conformability of the study findings, raw data, interview and observation notes, documents and records collected from the field, and others were documented for cross checking. To maintain the transferability of the finding, appropriate probes were used to obtain detailed information on responses, and study participants were selected based on their specific purpose to answer study questions and to get greater in-depth findings”. |
| **Comments from Reviewer #2** | |  |
| All comments have been addressed. | | Thank you so much dear reviewer for your valuable input for the improvement of our manuscript. |
