# Supplementary material for: Covid-19 vaccine uptake and its associated factors among adult population in Dangila district, Awi Zone, Northwest Ethiopia: A mixed method study
Source: PLoS One. 2024 May 14;19(5):e0302531. doi: 10.1371/journal.pone.0302531 (PMC11093357; doi:10.1371/journal.pone.0302531)
Supplement: Attachment — Submitted filename: A rebuttal letter 4.docx [file pone.0302531.s007.docx]

**A rebuttal letter 4**

Journal name: PLOS ONE

PONE-D-23-08920

**Title: Covid-19 vaccine uptake and its associated factors among adult population in Dangila district, Awi Zone, Northwest Ethiopia: A mixed method study**

| **Editor’s comments** | **Authors’ response** |
| --- | --- |
| **Journal Requirements:**  Please review your reference list to ensure that it is complete and correct. If you have cited papers that have been retracted, please include the rationale for doing so in the manuscript text, or remove these references and replace them with relevant current references. Any changes to the reference list should be mentioned in the rebuttal letter that accompanies your revised manuscript. If you need to cite a retracted article, indicate the article’s retracted status in the References list and also include a citation and full reference for the retraction notice. | Thank you dear editor for your valuable comment. The revised manuscript is revised for the presence of retracted references. We found that none of them are retracted. |
| **Comments from reviewer #1** | |
| 1. Authors are suggested to annex item by item description of knowledge and attitude questions so that readers would compare the cumulative reports in the respective tables of the result section. This same figures of knowledge and attitude reported after changing the analysis approach between revised version 2 and 3. This can be trusted when the item by item reportes are annexed. The second, comment is that the triangulations are not adequately done. Please pick beleifs and perceptions in the qualitative and triangulate them with quantitative knowledge and attitude sections. Do not report qualitative findings seeminly as separate section of the study. | - Thank you dear reviewer for your constructive comments which are too crucial for the improvement of our manuscript. - As per your comment, the item by item descriptions of knowledge and attitude questions have been uploaded as supplementary information file with the revised manuscript. - Regarding to the triangulation of the qualitative finding with quantitative one, we have adhered to your valuable comments. Beleifs and perceptions in the qualitative finding are not resented separately. We have triangulated it in the heading “Factors associated with **vaccine uptake**” (page 14, line 351-401). Here, factors identified by quantitative analysis have been triangulated with qualitative findings where beleifs and perceptions have been triangulated too. - The **Respondents’ Knowledge and Attitude characteristics described at** (page 12, line 315-323) is a purely descriptive finding where the qualitative findings of related terms shouldn’t be triangulated with. |
